# First person – Antonio Barral and Isabel Rollan

**DOI:** 10.1242/bio.049171

**Published:** 2019-12-02

## Abstract

First Person is a series of interviews with the first authors of a selection of papers published in Biology Open, helping early-career researchers promote themselves alongside their papers. Antonio Barral and Isabel Rollan are co-first authors on ‘*Nanog* regulates Pou3f1 expression at the exit from pluripotency during gastrulation’, published in BiO. Antonio is a PhD student in the lab of Miguel Manzanares at Centro Nacional de Investigaciones Cardiovasculares Carlos III (CNIC), Spain, investigating embryonic development, pluripotency and reprogramming. Isabel is a technical officer at the European Molecular Biology Laboratory (EMBL), Germany, in the lab of Alexander Aulehla, investigating gene editing, embryonic development and gene regulation.


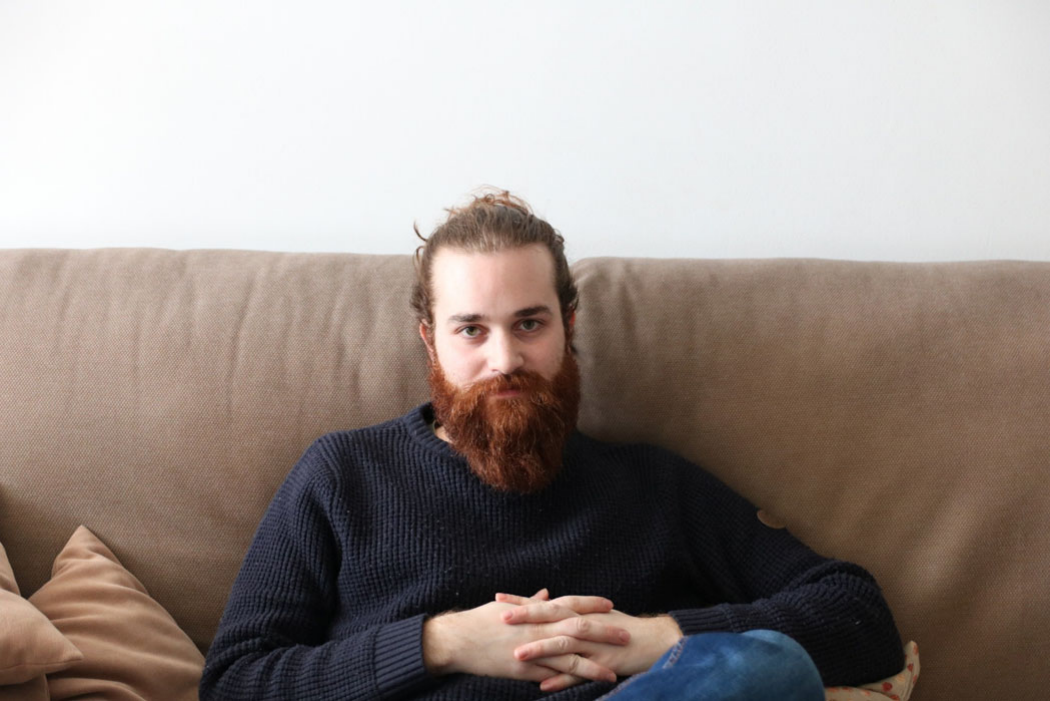


**Antonio Barral**


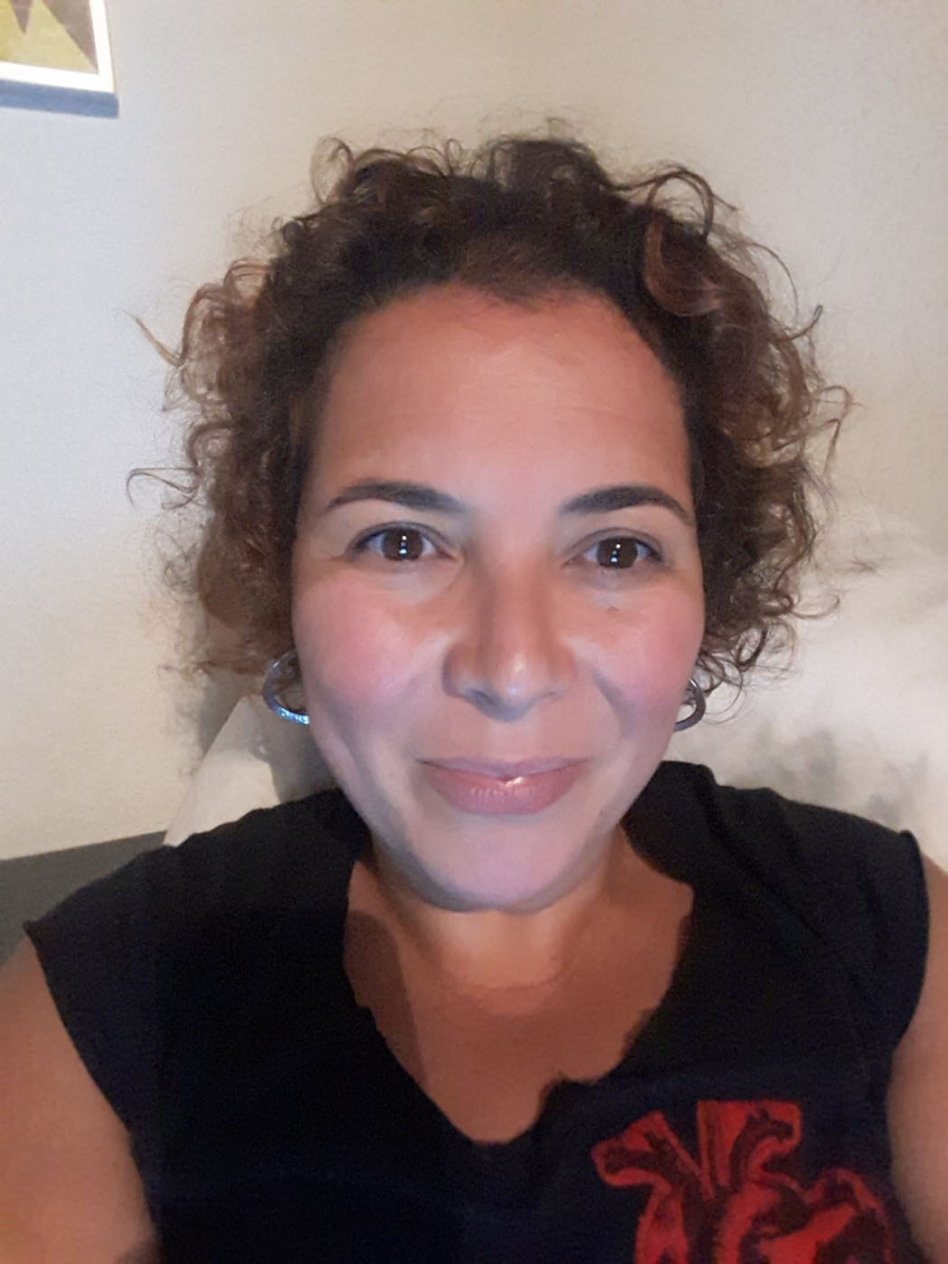


**Isabel Rollan**

**What is your scientific background and the general focus of your lab?**

**AB:** I studied biotechnology at Seville, and then I did a Master's in molecular biomedicine. I did my Master's thesis at my current lab, and I enjoyed working in the area of embryo development so much that I decided to carry on with my PhD with the very same group.

**IR:** I studied biology at the University of Seville and I did my thesis in toxicology at the same university. My involvement in science started with a focus on laboratory animal science. After several years working in different animal facilities, I moved to Miguel Manzanares' laboratory, the Functional Genomics Lab at the Spanish National Center of Cardiovascular Research (Madrid, Spain). There I performed several tasks, including the generation of genetically modified mouse lines, enhancer reporter assays, supporting the design of gene-targeting strategies (mainly by CRISPR-Cas9) and the development and application of tools for our daily work. After this exciting stay collaborating in different projects, I moved to my current work in the EMBL Transgenic Service (Heidelberg, Germany).

**How would you explain the main findings of your paper to non-scientific family and friends?**

**AB:** As most people know, we arise from a single cell. All the complexities and nuances we can find in an adult human being come from this very simple and unique cell: the zygote. Therefore, a very relevant question to tackle is how this takes place; what controls and directs such a well-orchestrated process starting with very limited building blocks? The first few cells that appear in an embryo have the potential to develop into any cell type present in the adult, but at some point during development they must lose this potential and choose a fate to follow; it is in this context where our publication comes into play. We have characterized how a protein (NANOG), which is widely known for being responsible for maintaining the cells from the early embryo in an undifferentiated or uncompromised state, does, surprisingly, participate in transitions away from said state.

**IR:** Apart from what my colleague Antonio has already described, I think that it is also interesting how a protein performs this regulation binding to a small region (just a few base pairs in length) in a non-coding region far away from its target gene. They can modulate the expression of that gene acting as interrupters that switch on or off their expression. This is not new, but here in the paper we show that we can work in transient to identify peaks that are important for that regulatory cascade. Despite us having generated the mouse line to confirm our results, the transient approach could be interesting to test several candidates. You could save time in the generation of mouse lines and still get the same information.

**What are the potential implications of these results for your field of research?**

**AB:** The appearance and maintenance of pluripotency has been very well characterized in the last few years, but we have seen how this focus has shifted towards understanding the steps necessary to escape from this state and what drives cell fate choices during early embryonic development. In this context, our findings stress the importance of taking into account the functional duality some factors can show: participating in the generation of pluripotency does not exempt a certain regulator from having functions that could seem conflicting, that is, specifically regulating steps later in development when pluripotency itself has already disappeared.

**IR:** This could be so-called ‘cell economy’; cells save resources, reusing a variety of different factors in different times and for different purposes.

“Participating in the generation of pluripotency does not exempt a certain regulator from having functions that could seem conflicting.”

**What has surprised you the most while conducting your research?**

**AB:** The idea that very rarely is there a single-purpose tool in any given organism. Reusing, adapting and repurposing pre-existing mechanisms is inherent to nature.

**IR:** I was more involved in the CRISPR part and for me it was surprising to see how deletions in transient can give you enough information about the importance of that region.

**Figure:**
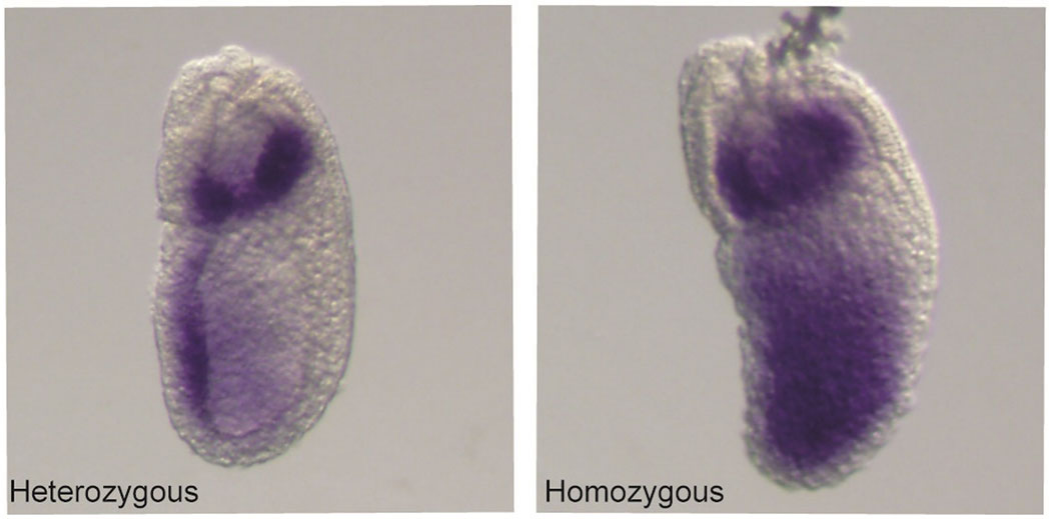
***Pou3f1* whole-mount *in situ* hybridization images of mouse embryos at embryonic day 7.5, either heterozygous for the deletion of the NANOG binding site (left) or homozygous (right).** An expansion of the expression domain of *Pou3f1* can be seen when the binding site is deleted from both alleles

**What, in your opinion, are some of the greatest achievements in your field and how has this influenced your research?**

**AB:** Clearly the rise of CRISPR. It has made things so much easier when interrogating the genome, it is now hard to think of projects that would not use it. Truly, having Isabel working hand-in-hand with me, with her vast CRISPR expertise, has been a blessing.

**IR:** Totally agree. The use of CRISPR is one of the greatest achievements in modern biological sciences. It has allowed us to create very fine deletions or modifications and create models for any disease. Besides, it's simple to use, fast and cheap (compared to other approaches used in the past).

“The use of CRISPR is one of the greatest achievements in modern biological sciences.”

**What changes do you think could improve the professional lives of early-career scientists?**

**AB** and **IR:** At least within our country of origin (Spain), we feel like the amount of scholarships offered to carry out PhDs and post-docs should be much greater, and the retribution fairer, considering the effort required. We also find the current pressure towards high-impact publishing very detrimental for the values intrinsic to scientific research, as in order to be in a position from which you can deliver good science, you need to be constantly pushing your results and resources to the limit. This is, from our point of view, very dangerous, generating a never-ending cycle that undermines the purpose of science itself.
